# Polygonatum sibiricum polysaccharide inhibits osteoporosis by promoting osteoblast formation and blocking osteoclastogenesis through Wnt/β-catenin signalling pathway

**DOI:** 10.1038/srep32261

**Published:** 2016-08-24

**Authors:** Li Du, Meng-Ni Nong, Jin-Min Zhao, Xiao-Ming Peng, Shao-Hui Zong, Gao-Feng Zeng

**Affiliations:** 1 Department of Spine Osteopathia, the First Affiliated Hospital of Guangxi Medical University, Nanning 530000, Guangxi, P. R. China; 2 College of Public Hygiene of Guangxi Medical University, Nanning 530000, Guangxi, P. R. China; 3 Department of Osteopathia, the First Affiliated Hospital of Guangxi Medical University, Nanning 530000, Guangxi, P. R. China; 4 Research Centre for Regenerative Medicine and Guangxi Key Laboratory of Regenerative Medicine, Guangxi Medical University, Nanning 530000, Guangxi, P. R. China

## Abstract

Bone homeostasis is maintained by a balance between bone formation by osteoblasts and bone resorption by osteoclasts. Osteoporosis occurs when osteoclast activity surpasses osteoblast activity. Our previous studies showed the plant-derived natural polysaccharide (Polygonatum sibiricum polysaccharide or PSP) had significant anti-ovariectomy (OVX)-induced osteoporosis effects *in vivo*, but the mechanisms of PSP’s anti-osteoporosis effect remains unclear. In this study, we assessed PSP’s effect on the generation of osteoblast and osteoclast *in vitro*. This study showed that PSP promoted the osteogenic differentiation of mouse bone marrow stromal cells (BMSCs) without affecting BMPs signaling pathway. This effect was due to the increased nuclear accumulation of β-catenin, resulting in a higher expression of osteoblast-related genes. Furthermore, the study showed PSP could inhibit the receptor activator of nuclear factor-κB ligand (RANKL)-induced osteoclastogenesis and exert prophylatic protection against LPS-induced osteolysis *in vivo*. This effect was also related to the increased nuclear accumulation of β-catenin, resulting in the decreased expression of osteoclast-related genes. In conclusion, our results showed that PSP effectively promoted the osteogenic differentiation of mouse BMSCs and suppressed osteoclastogenesis; therefore, it could be used to treat osteoporosis.

Osteoporosis, a type of bone metabolic disease characterised by low bone mineral density and deterioration of the bone micro-architecture, is a growing public health problem. It can lead to an increased risk of fragility fractures. Osteoporosis is a predominantly age-related disease, affecting postmenopausal women in particular. This disease puts these women at a greater risk of fragility fractures during the remainder of their lifetime^1^. Fractures caused by osteoporosis result in significant morbidity and mortality and put an increased economic burden on society^2^.

Various drugs in clinical trials have been effective in treating or preventing osteoporosis. These drugs mostly include two categories: 1. bone absorption-inhibitor drugs (e.g., oestrogen, selective oestrogen receptor modulators and bisphosphonate); 2. bone formation-acceleration drugs (e.g., PTH); however, potential adverse side effects were observed, particularly regarding hormone replacement therapy drugs and bisphosphonates^3^,^4^. Therefore, new drug options from natural resources are in great demand. Several natural compounds, including Genistein, Icariin, Naringin and Resveratrol, have presented anti-osteoporosis properties^5^,^6^,^7^,^8^. PSP is isolated from *Polygonatum sibiricum*, which could have anti-inflammatory characteristics^9^ and attenuate amyloid-β-induced neurotoxicity^10^; however, there is little research on PSP’s effect on osteoporosis.

During adult life, osteoprogenitors originate from bone marrow mesenchymal stem cells^11^. Hormonal and local factors affect the osteoblastic differentiation program of BMSCs^12^, including those involved in the canonical Wnt/β-catenin signalling. The increased nuclear accumulation of β-catenin has a significant role in promoting osteoblast differentiation and bone formation^13^. In addition, the increased nuclear accumulation of β-catenin also inhibits osteoclast differentiation^14^.

In our previous study, we found that bone loss was reversed and osteoporosis was prevented *in vivo* by administrating PSP to ovariectomized rats^15^; however, the mechanisms of PSP’s anti-osteoporosis effects remain unclear. In this study, we tried to assess the effects of PSP on the generation of osteoblast and osteoclast *in vitro*, and initially explore whether Wnt/β-catenin pathway was required in the therapeutic action of PSP on osteoporosis.

## Results

### Cell growth and morphological changes

The morphology of normal BMSCs were long, spindle-shaped, fibroblast-liked. Because of vigorous cell proliferation and a shorter growth incubation period, cells could be covered with a single layer in 2–3 days. After induced osteogenesis for 3 days, the BMSCs appeared variable in cell volume with a short spindle shape. For 6–7 days, most of the cells showed the uniform polygon shape with abundant micro-particles in the cytoplasm. In the process of osteogenesis, a multilayer growth of cells was formed. The cells in high-density areas gradually transformed towards osteoblasts, along with increased secretion of extracellular matrix.

### PSP promoted osteogenesis

**Figure 1:**
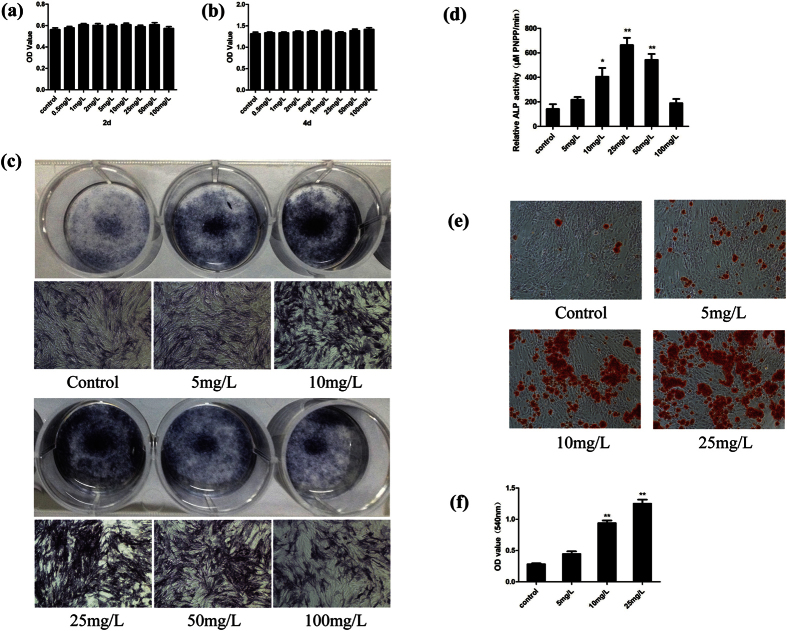
PSP enhanced the osteogenic differentiation of BMSCs. (**a**,**b**) The OD values of PSP-treated BMSCs were tested by MTT assays at 2d and 4d. There were no statistical significance among these groups. (**c**) After BMSCs had been cultured with OBM + PSP (0, 5, 10, 25, 50, 100 mg/L) for 7 days, they were identified by alkaline phosphatase staining. The cells treated with PSP (10 mg/L, 25 mg/L, 50 mg/L) were stained dark blue. (**d**) The activity of ALP of BMSCs. Compared to untreated control group, the group treated with PSP (10 mg/L, 25 mg/L, 50 mg/L) could significantly increased the activity of ALP, which was consistent with the results of ALP staining, as mentioned above. (**e**,**f** ) Mineralized nodules were detected using Alizarin Red S staining. More plaques of calcified extracellular matrix could be detected on day 28 at 10 mg/L and 25 mg/L PSP. The OD value of solutes of Mineralized nodules (PSP 10 mg/L and 25 mg/L) was significantly higher than untreated control group. The date is showed as the mean ± S.E. *p < 0.05 or **p < 0.01 vs. control value.

With the effect of osteogenic differentiation media (OBM), the BMSCs could differentiate into osteoblasts. Several stages were needed in the process of bone formation, including expansion of osteoprogenitors, maturation of extracellular matrix and deposition of minerals in the matrix. We firstly tested the cell proliferation or cytotoxicity effects of PSP on BMSCs. The OD values showed there were no inhibitory or cytotoxic effects of PSP at doses (5–100 mg/L) (Fig. 1a,b). The effect of PSP on alkaline phosphatase (ALP) expression, which is an early marker for osteoblastic activity, was observed. 10, 25 and 50 mg/L PSP significantly induced the expression of ALP in the BMSCs on day 7, compared with the untreated control (Fig. 1c,d). To investigate PSP’s effect on depositing minerals in the matrix, we observed the number of minerals by Alizarin red S staining. More plaques of calcified extracellular matrix could be detected on day 28 at 10 mg/L and 25 mg/L PSP, compared with the untreated control (Fig. 1e,f).

### PSP enhanced osteoblastic gene expression

**Figure 2:**
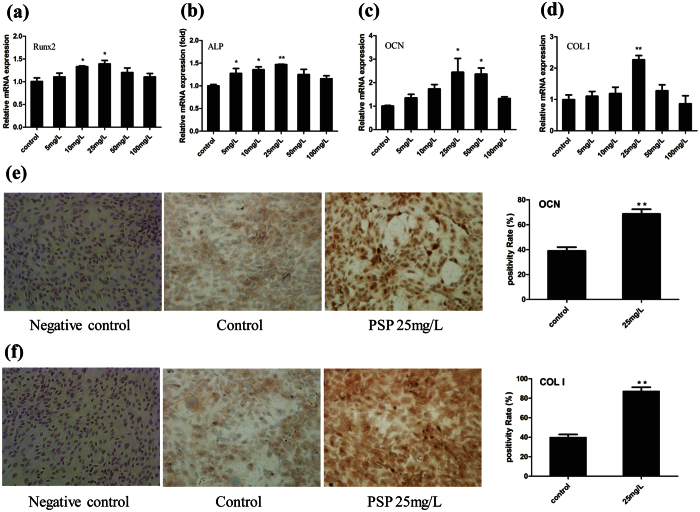
PSP enhanced osteoblastic gene expression. (**a**–**d**) Effects of different concentrations of PSP on the mRNA expression of Runx2(7d), ALP(7d), and OCN (21d), COL I(7d) of BMSCs. β-actin served as an internal control. The date is showed as the mean ± S.E. *p < 0.05 or **p < 0.01 vs. control value. (**e**,**f**) Comparison of the expression of COL I(7d) and OCN(21d) between the group treated with PSP (25 mg/L) and control group in the osteogenic differentiation of BMSCs by cell immunohistochemical staining (*p < 0.05, **p < 0.01).

To further confirm the effect of PSP on the promotion of osteogenic differentiation of the BMSCs, we observed the activation on the mRNA levels of bone differentiation marker genes, including COL I (7), ALP (7d), Runx2 (7d) and Osteocalcin (OCN 21d). Compared to the untreated control, the treated groups with PSP (5–50 mg/L) demonstrated higher gene expression (Fig. 2a–d). Cell immunohistochemistry analysis also revealed that the PSP (25 mg/L) increased the expression of COL I (7d), OCN (21d) (Fig. 2e,f). These above results suggested that PSP promoted osteogenic differentiation of the BMSCs *in vitro*.

### PSP suppressed osteoclastogensis

**Figure 3:**
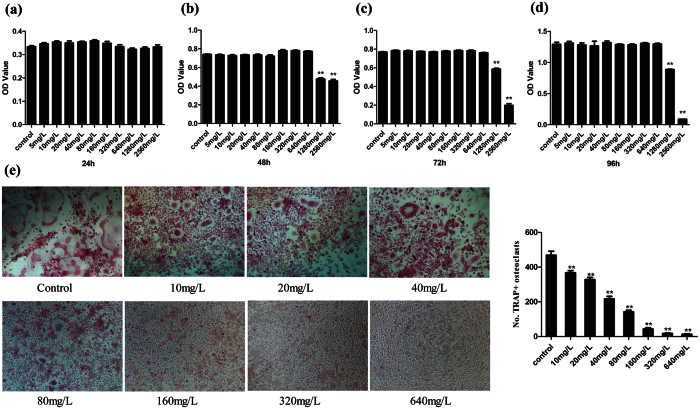
PSP inhibited RANKL-induced osteoclast differentiation without cytotoxic effects on BMMs *in vitro*. (**a**–**d**) The OD values of PSP-treated BMMs were tested by CCK-8 assays at 24, 48, 72 and 96 h. The values in 1280 mg/L and 2560 mg/L PSP groups were significantly decreased compared with the control group (**P < 0.01). (**e**) BMMs were treated with various concentrations of PSP in the presence of M-CSF (30 ng/mL) and RANKL (50 ng/mL) for 6 days. Then, the TRAP staining assay was performed. The number of TRAP-positive multinuclear osteoclasts was significantly deceased in a dose-dependent manner(n = 3, **P < 0.01).

Before evaluating whether PSP could suppress the osteoclast differentiation of BMMs, it was required to have no cytotoxicity *in vitro*. Therefore, we designed 10 different concentrations of PSP (0, 5, 10, 20, 40, 80, 160, 320, 640, 1280 and 2560 mg/L) for 24, 48, 72 and 96 hours to determine which doses inhibited cell proliferation. The OD values in 1280 mg/L and 2560 mg/L PSP groups decreased significantly compared with the control group. There were no inhibitory or cytotoxic effects of PSP at 640 mg/L and below (Fig. 3a–d). To determine the effect of PSP on RANKL-induced osteoclast differentiation, we treated BMMs in the presence of RANKL and M-CSF with 0, 10, 20, 40, 80, 160, 320 and 640 mg/L of PSP. We counted TRAP stain-positive multinuclear osteoclasts. The TRAP-positive cell number (No. nuclei ≧3) decreased from 468.3/well (control), 217.0/well (PSP 40 mg/L) to 12.7/well (PSP 640 mg/L) (Fig. 3e).

### PSP inhibited osteoclast specific gene expression

**Figure 4:**
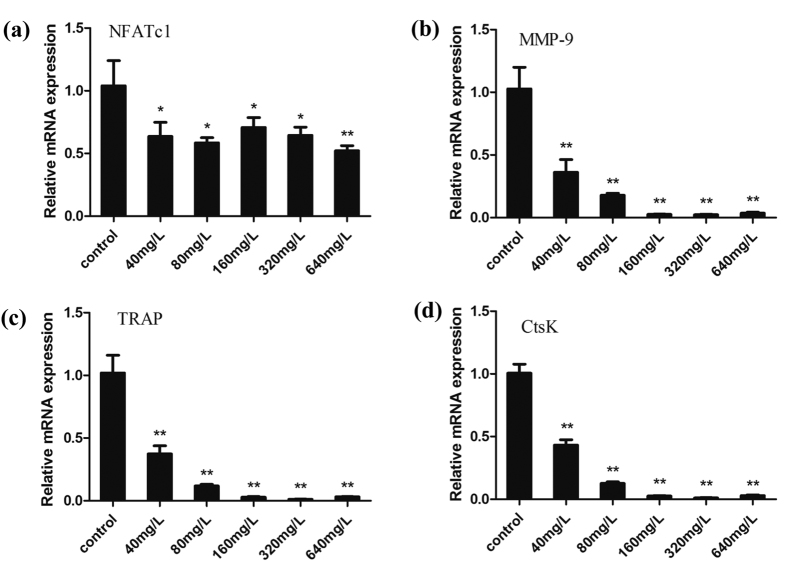
PSP inhibited osteoclast specific gene expression. (**a**–**d**) Effects of different concentrations of PSP on the mRNA expression of osteoclast-related genes (NFATc1, MMP-9, TRAP andCtsK) in BMMs. Total RNAs were extracted from BMMs treated with PSP on day 6 to perform quantitative RT-PCR for osteoclast differentiation markers. β-actin served as an internal control. The date is showed as the mean ± S.E. *p < 0.05 and **p < 0.01. PSP inhibited osteoclast specific gene expression (MMP-9, TRAP andCtsK) in a dose-dependent manner.

In addition, the mRNA expressions of osteoclast-specific genes, including TRAP, MMP-9, CtsK and NFATc1, were significantly down-regulated in a dose-dependent manner during the RANKL-induced osteoclast formation after PSP treatment (Fig. 4a–d). Collectively, these results showed that PSP had inhibitory effects on osteoclastogenesis.

### PSP regulated β-catenin in both osteoblasts and osteoclasts

**Figure 5:**
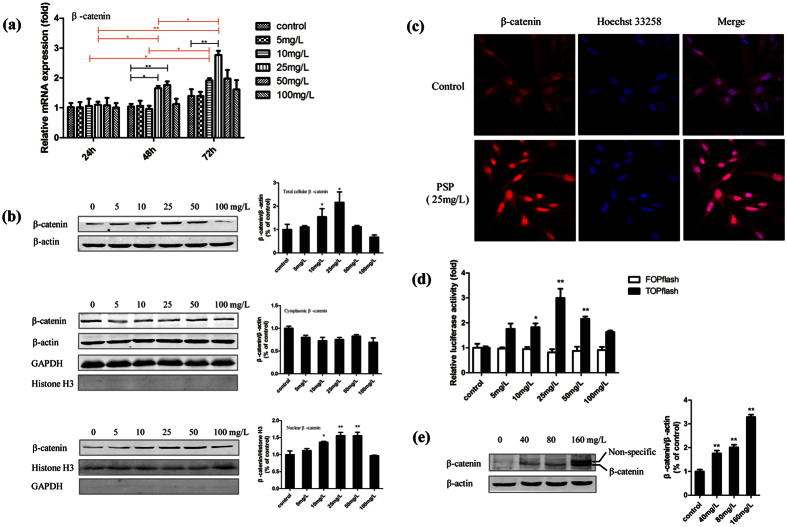
PSP regulated the expression of β-catenin in both osteoblasts and osteoclasts. (**a**) Effect of PSP on β-catenin mRNA expression in the process of osteoblast differentiation. After BMSCs were treated with various concentrations of PSP for 24 h, 48 h and 72 h, total RNAs were extracted to detect β-catenin. The red line represents that the comparison of level of β-catenin mRNA expression in the same group at different time. The black line represents that the comparison of level of β-catenin mRNA expression in different groups at the same time (*P < 0.05, **P < 0.01). (**b**) BMSCs were treated with various concentrations of PSP for 72 h and subjected to Werstern Blot to analyze the expression of β-catenin (n = 3, *P < 0.05, **P < 0.01). β-actin and Histone H3 were used as the internal reference protein for total (cytoplasmic) and nuclear extracts respectively. The level of GAPDH and Histone H3 in both the cytoplasmic and nuclear fractions were showed to evaluate effectiveness of the separation of cytoplasmic and nuclear proteins. (**c**) BMSCs were treated with 25 mg/L PSP for 72 h, and immunofluorescence was performed to detect β-catenin nuclear translocation (red). DAPI (blue) was showed the position of the nuclei. Immunofluorescence staining showed that PSP treatment could urge BMSCs to accumulate β-catenin in the nucleus (magnification 40×, oil lens). (**d**) PSP promotes β-catenin-mediated gene transcription. BMSCs were transientlyco-transfected with 100 ng TOPFlash or FOPFlash and 20 ng pRL-TK. The date is showed as the mean ± S.E. Black bars represent TOPFlash luciferase activity increased multiple-fold, however, white bars, as FOPFlash luciferase activity, have no significantly changes (*P < 0.05, **P < 0.01). (**e**) BMMs were treated with various concentrations of PSP for 72 h and subjected to Werstern Blot to analyze the expression of β-catenin (n = 3, **P < 0.01). β-actin was used as the internal reference protein.

It is known that a Wnt/β-catenin signalling pathway plays an important role in the induction process of bone formation. As a transcriptional co-activation factor, the β-catenin protein is a key to activating the classic Wnt/β-catenin signalling pathway. In this paper we employed qRT-PCR and WB techniques to detect the changes in the expression levels and subcellular distribution of β-catenin. Immunofluorescence labelling was performed to evaluate the transfer and localisation of β-catenin in cells, confirming that PSP raised expression and activation of β-catenin and promoted it into the nucleus. In this study, cellular total RNAs were extracted for detecting β-catenin mRNA expression when the BMSCs were treated with PSP for 24, 48, and 72 hours. The results showed that the levels of β-catenin mRNA expression at concentrations of 10 mg/L and 25 mg/L increased along with extending the induction time (Fig. 5a). The level of β-catenin mRNA expression at concentration of 25 mg/L was significantly higher than the control group at 48 hours and 72 hours (Fig. 5a). After being treated with PSP for 72 hours, the expression levels of β-catenin in total protein extracts, nuclear and cytoplasmic extracts were detected by WB. The results showed that PSP treatment at 5–50 mg/L could significantly increase the total and nuclear amount of β-catenin in a dose-dependent manner, peaking at 25 mg/L, in accordance with the β-catenin mRNA expression level. The β-catenin level in cytoplasmic protein extracts, however, had no obvious difference between groups (Fig. 5b). To further confirm the effects of PSP on the Wnt/β-catenin signalling pathway in the BMSCs, we used immunofluorescence labelling to detect the distribution of β-catenin after being induced for 72 hours. The results showed that compared with the control group, the 25 mg/L PSP group appeared to accumulate the β-catenin in the cytoplasm and especially in the nucleus (Fig. 5c). It revealed that PSP could promote the accumulation of β-catenin in the nucleus, so as to activate the Wnt/β-catenin signalling pathway.

With the accumulation of β-catenin in the nucleus, it interacts with transcription factors TCF/LEF and then activates the expression of downstream target genes of Wnt/β-catenin signalling pathway. TOPFlash plasmid, containing the firefly luciferase open-reading frame and TCF/LEF binding sites in its upstream promoter sequences, is regulated by the β-catenin activity. To test whether PSP may promote the activation of downstream target genes on the Wnt/β-catenin signalling pathway, we transiently co-transfected the BMSCs with TOPFlash or FOPFlash along with pRL-TK. After being transfected for 6–8 hours, the cells were treated with various concentrations of PSP, and the luciferase activity was detected at 48 hours post-treatment. The results showed that the PSP treatment groups (10, 25, 50 mg/L) significantly enhanced TOPFlash luciferase activity compared with the control group (of which the 25 mg/L group had a maximum effect, increased by 3 times) (Fig. 5d). FOPFlash luciferase activity, however, had no significant changes. The above results showed that PSP could enhance the transcriptional activity of β-catenin/TCF and activate the expression of downstream target genes.

The above mentioned results confirmed PSP could increase the total amount of β-catenin in the osteoblast differentiation process of BMSCs. Therefore, we assumed that osteoclast differentiation also had a direct relationship with the Wnt/β-catenin signalling pathway. We used WB to detect the expression level of β-catenin in total proteins extracted from BMMs after PSP treatment for 72 hours. As shown in Fig. 5e, PSP treatment could significantly increase the total amount of β-catenin in a dose-dependent manner (Fig. 5e). We speculate dual band may be caused by the phosphorylation of β-catenin.

### PSP didn’t affect BMPs signaling pathway in osteoblastic differentiation of BMSCs

**Figure 6:**
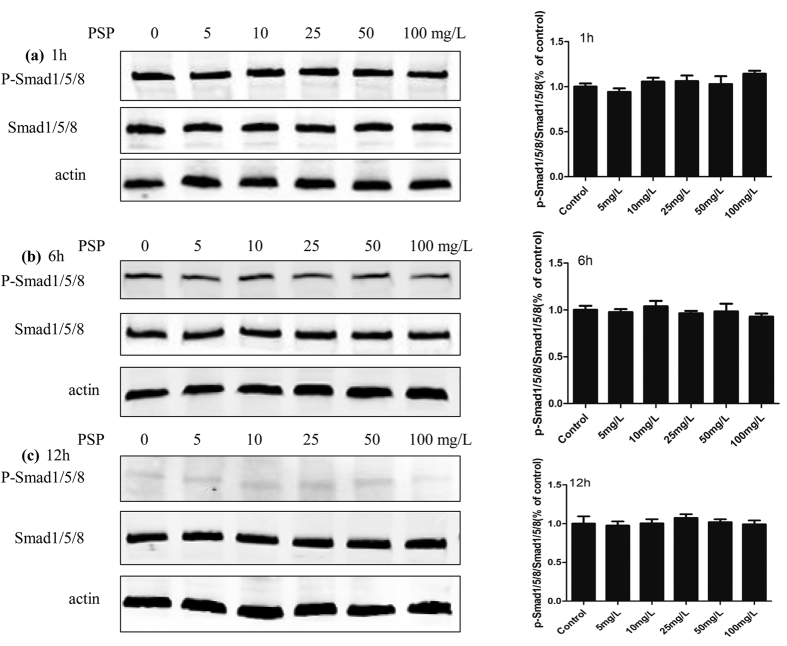
PSP didn’t affect BMPs signaling pathway in osteoblastic differentiation of BMSCs. (**a**–**c**) BMSCs were treated with OBM and PSP(0 mg/L, 5 mg/L, 10 mg/L, 25 mg/L, 50 mg/L, 100 mg/L). Protein expression of p-Smad1/5/8 was detected at 1 h, 6 h and 12 h. No statistical difference was observed among these groups.

BMPs play an important role in the formation and remodeling of bone by stimulating the differentiation of osteoblast cells^16^. The action of BMPs is mediated by downstream transcription factors Smad1/5/8. Western blot analysis was performed to detect protein expression of p-Smad1/5/8, and to provide information regarding the possible mechanism. BMSCs were treated with OBM and PSP(0 mg/L, 5 mg/L, 10 mg/L, 25 mg/L, 50 mg/L, 100 mg/L). We detected protein expression of p-Smad1/5/8 at 1 h, 6 h and 12 h. No statistical difference was observed on the protein expressions of p-Smad1/5/8 among the different concentrations of PSP groups(0 mg/L, 5 mg/L, 10 mg/L, 25 mg/L, 50 mg/L, 100 mg/L) (Fig. 6a–c). These results suggested that PSP promoted osteoblastic differentiation without affecting BMPs signaling pathway.

### PSP prevented LPS-induced osteolysis

**Figure 7:**
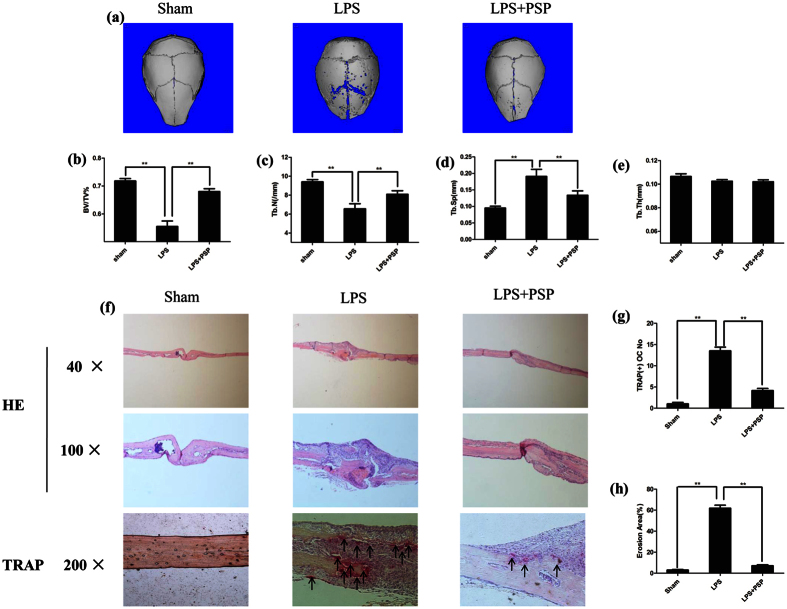
PSP prevented LPS-induced osteolysis. (**a**) Representative micro-CT 3D reconstructed images were obtained for each group. (**b**,**c**,**d**,**e**) Quantitative analysis of bone volume/tissue volume (BV/TV), trabecular number (Tb.N.), trabecular separation (Tb.Sp.) and trabecular thickness (Tb.Th.) (**P < 0.01). (**f** ) Representative images of decalcified bone stained with H&E and TRAP from sham, LPS and LPS+PSP Groups. (**g**,**h**) The number of TRAP positive multinucleated osteoclasts (≥3 nuclei) on the bone surface erosion area (the percentage ofinfiltration fibrotic area against total tissue area) was measured (**P < 0.01).

To determine the preventive effect of PSP on LPS-induced osteolysis, mice received local subcutaneous injections of PBS, LPS or LPS with PSP onto the sagittal suture of calvarium for 7 days. One day before LPS injection (prophylactic treatment), the LPS with PSP and PBS groups were injected with PSP or PBS, respectively. Seven days after LPS injection, three-dimensional micro-computed tomographic analysis revealed PSP treatment protected the mice from LPS-induced osteolysis, as shown by increased BV/TV (bone volume/tissue volume) and Tb.N (trabecular number) in treated mice relative to the mice without PSP treatment (Fig. 7a–c). Furthermore, Tb.Sp (trabecular separation) was significantly reduced in the LPS + PSP group when compared with LPS control group (Fig. 7d). To further confirm the preventive effect of PSP on LPS-induced osteolysis, histomorphometric analysis was also performed. The TRAP^+ve^ (tartrate-resistant acid phosphatase) OC No and Erosion Area(%) were decreased in PSP treated mice as compared with LPS group (Fig. 7f–h). Thus our data demonstrated that prophylactic treatment with PSP could protect LPS-induced osteolytic bone loss *in vivo*.

## Discussion

The development of natural plant extracts has provided a viable option for the treatment of osteoporosis. PSP is isolated from *Polygonatum sibiricum*, which is an effective remedy for inflammation and amyloid-β-induced neurotoxicity^9^,^10^. In our previous study, we found that PSP reversed bone loss and prevented osteoporosis *in vivo*^15^. In this study, we found that PSP had the ability to promote osteoblast formation and inhibit osteoclast formation probably through Wnt/β-catenin pathway.

In this report, we primarily explored the effect of PSP on osteoblast formation. Our results showed that PSP significantly enhanced the osteogenic differentiation of BMSCs at concentrations ranging from 5 to 50 mg/L. We found that PSP promoted osteogenic differentiation by increasing the activity of ALP and the expressions of osteoblastic differentiation makers, such as ALP, COLI, Runx2, and Osteocalcin. The activity of ALP is often used as a marker of early osteogenic differentiation^17^,^18^. Therefore, we initially explored the proper concentrations of PSP according to the activity of ALP. Our results revealed that PSP at 10, 25 and 50 mg/L significantly induced the expression of ALP in a dose-dependent manner without affecting the viability of the BMSCs. During the process of BMSCs differentiation to osteoblasts treated with PSP, the BMSCs showed distinct expressions of osteoblast-related genes, including ALP, COL I, Runx2, and OCN, compared to the untreated group. Runx2 is the specific transcription factor of osteoblasts^19^,^20^ and can play a leading role in the process of osteogenic differentiation by regulating the expression of ALP, COL I and OCN genes. Collagen I is another bone marker; its role is to act as a substrate for the calcium deposition and cell adhesion^21^. OCN is a kind of non-collagenous protein and can combine with calcium and hydroxyapatite, which is necessary for normal bone formation. The expression of the OCN gene gets turned on in the early stages of calcification and reaches the highest spot when generating mature mineralisation nodes; therefore, OCN is the characteristic protein of osteoblasts and the major sign of the start of mineralisation^22^. Considering the above results, we have reasons to believe that PSP stimulates osteogenesis of BMSCs.

The Wnt/β-catenin signalling pathway is considered as the most classic and clear signalling pathway; it is an extremely conservative signalling pathway involved in biological evolution^23^,^24^,^25^. In recent years, many studies have shown that the Wnt/β-catenin signalling pathway is closely related to the stimulation of osteogenic differentiation, bone formation and the prevention of osteoporosis^26^,^27^. The role of the Wnt/β-catenin signalling pathway is critical for mammalian BMSCs in the process of osteogenesis^28^,^29^,^30^. Thus, an idea of studying the activation of canonical Wnt/β-catenin signalling in BMSCs has been generated.

Extracellular Wnt signalling proteins are used in combination with a plasma-membrane receptor to activate the classic Wnt/β-catenin signalling pathway. As a result, β-catenin stabilises within the cytoplasm, accumulates into the nucleus and interacts with TCF/LEF to activate the expression of Wnt pathway downstream target gene. In the process of osteogenic differentiation, β-catenin is a key mediator and plays a crucial role in the Wnt signalling pathway. This leads us to detect the expression of β-catenin after being treated with PSP.

In this paper, the activation of Wnt signalling pathway in BMSCs was tested. The results demonstrated that the PSP dose dependently enhanced the expression level of β-catenin in the nucleus and stimulated its transcriptional target genes. Our results were similar to the recent findings that Wnt/β-catenin signalling contributed to the acceleration of osteoblastic differentiation *in vitro*^31^,^32^. Our study showed that PSP treatment led to an increased level of β-catenin without affecting BMPs signaling pathway. Still, it cannot be ruled out that PSP treatment may also stimulate other action targets or activate other signalling pathways to promote bone formation. Thus, there needs to further explore other action targets for the effects of PSP on osteoblastic differentiation in BMSCs.

In addition to promoting the osteoblastic differentiation, PSP also inhibits the RANKL-induced osteoclast differentiation on BMMs. Our results showed that PSP inhibited RANKL-induced osteoclastogenesis at concentrations of 40–640 mg/L without affecting the viability of BMMs. Mature osteoclast formation was affected at these doses, resulting in significant reductions in the number of multinuclear and TRAP stain positive cells and the expression of osteoclast characteristic genes (MMP-9, NFATc1, TRAP, and Ctsk). Our results firstly showed that PSP could preserve bone mass in an LPS-induced osteolysis model. Thus, our study *in vivo* and vitro identified PSP as a potential new drug for the treatment of osteoporosis. In this study, we also found that PSP treatment could significantly increase the expression level of β-catenin in a dose-dependent manner. We speculate that the inhibiting effect of PSP on osteoclast differentiation may be due to the activation of canonical and non-canonical cAMP/PKA pathways, which can activate the crucial transcription factor NFATc1 in osteoclast progenitors^33^,^34^. However, until now, we have not been able to determine the signalling way (canonical or non-canonical Wnt pathway) through which PSP inhibits osteoclastogenesis. Therefore, further research needs to be conducted about the inhibition mechanism of PSP on the differentiation of osteoclasts.

Taken together, our results demonstrate that PSP could treat osteoporosis by promoting osteoblast formation and inhibit osteoclastogenesis through up-regulation of the Wnt/β-catenin signalling pathway. However, the underlying mechanisms are not completely understood. For prevention and treatment of osteoporosis, further studies are still required to explore more specific mechanisms.

## Methods

This project fully considered and protected the rights and interests of the study objects. The use of animals in experiments was approved by relevant authority (The Ethics Committee of the first affiliated hospital of Guangxi medical university). All experimental procedures were conducted in conformity with institutional guidelines for the care and use of laboratory animals in the first affiliated hospital of Guangxi medical university, China. It meets the criteria of Ethical Review Committee.

### BMSC culture and preparation of osteoblast medium

The BMSCs were cultured in L-DMEM supplemented with 10% foetal bovine serum, 100 U/ml penicillin and 100 mg/ml streptomycin at 37 °C in a 5% CO_2_-humidified atmosphere. They were passaged at a ratio of 1:6 plates when the cells grew to 80–90% confluence. For osteogenic differentiation, the BMSCs were cultured with OBM containing 10% FBS, 100 U/ml penicillin, 100 mg/ml streptomycin, 50 μg/ml ascorbic acid, 10 mM sodium β-glycerophosphate and 10^−7^ M dexamethasone in L-DMEM^35^,^36^. Afterwards, the PSP (0, 5, 10, 25, 50 and 100 mg/L) was added to the OBM and replaced once every 2–3 days. The change of cell morphology and the cellular growing state were observed using an inverted microscope.

### MTT assay

The cell proliferation effects of PSP on the BMSCs were determined using the MTT assay. The BMSCs were plated into 96-well plates at a density of 9 × 10^3^ cells/well in triplicate in DMEM supplemented with 10% FBS and 1%penicillin. After 24 hours, the cells were treated with different concentrations of PSP (0, 5, 10, 25, 50, 100 mg/L). The MTT assay was measured at Day 2, Day 4 after the treatment. In this assay, MTT performed Formazan crystals by the mitochondrial dehydrogenase of viable cells. The optical density (OD) of the formazan solution was read on Microplate Reader (Thermo Multiskan GO, USA) at 540 nm.

### ALP staining and activity assay

The BMSCs were seeded into 12-well plates at a density of 2 × 10^4^ cells/cm^2^ in OBM and were treated with various concentrations of PSP for 7 days with a medium change every 2–3 days. After treatments, the cells were fixed with 4% paraformaldehyde for 30 minutes and stained with 1 mL BCIP/NBT (Beyotime Biotechnology, Shanghai, China) for 30 minutes at room temperature in the dark. The staining solution was then abandoned, and the cells were washed with phosphate-buffered saline (PBS) and photographed using an inverted microscope with a Nikon digital camera (TS100-F).

For the ALP activity assay, the cells were washed twice with ice-cold PBS and lysed with ice-cold 0.1% Triton X-100/PBS overnight at 4 °C. The supernatants were collected in new Eppendorf tubes from the cell lysates, which were centrifuged at 4 °C 12000 rpm for 5 minutes, and were kept on ice until assayed. According to the ALP assay kit (Beyotime Biotechnology, Shanghai, China) manufacturer’s suggested instructions, the PNPP substrate solution was mixed with the collected supernatant into a 96-well plate at 37 °C for 10 minutes, and the reaction was stopped by NaOH solution. The OD values at the wavelength of 405 nm were measured by using Microplate Reader (Thermo Multiskan GO, USA), and the ALP activity was normalised by total protein concentration, which was determined with the BCA method. Each group was performed in triplicate wells; the averages were then taken and repeated three times.

### Alizarin Red Staining

To investigate the effect of PSP on osteoblast differentiation, the BMSCs were seeded at a density of 2 × 10^4^ cells/cm^2^ into 6-well plates that had been pre-coated with 0.1% gelatin solution. After treatment for 28 days with different concentrations of PSP, the cells were gently washed twice with PBS, then fixed with 4% paraformaldehyde for 20 minutes and stained with a 1-mL/well Alizarin red S solution (Cyagen Biosciences, Guangzhou, China) for 5 minutes at 37 °C. The images of extracellular matrix mineralisation nodules were captured using an inverted microscope with a digital camera. After photographing the nodules, the Alizarin red S-stained mineralisation nodules were incubated with a 1-mL/well, 100 mM cetylpyridinium chloride (CPC) for 1 hour to destain and extract calcium-bound Alizarin red into the solution. The OD values at 550–570 nm were obtained by an enzyme-labelled instrument.

### BMMs isolation and osteoclast differentiation

The primary BMMs were isolated from the femoral and tibial bone marrow of a 7-week-old male, C57BL/6 mouse, and cultured in L-DMEM supplemented with 10% FBS, 100 U/ml penicillin and 100 mg/ml streptomycin at 37 °C in a 5% CO_2_-humidified atmosphere until reaching 90% confluence^37^. The cell proliferation effects of PSP on the BMMs were determined using the CCK-8 assay. The BMMs were plated into 96-well plates at a density of 9 × 10^3^ cells/well in triplicate in a complete medium containing 30 ng/mL M-CSF. After 24 hours, the cells were treated with different concentrations of PSP (0, 5, 10, 20, 40, 80, 160, 320, 640, 1280 and 2560 mg/L) for 24, 48, 72 or 96 hours. Then, 10 μL of CCK-8 buffer (Dojindo Molecular Technology, Kumamoto, Japan) was added to each well, and plates were incubated for an additional 2 hours. The OD values at the wavelength of 450 nm were measured by using Microplate Reader. After an analysis of the proliferation effects, the BMMs were differentiated into osteoclasts in the presence of 30 ng/mL M-CSF, 50 ng/mL RANKL and different concentrations of PSP (0, 40, 80, 160, 320, and 640 mg/L). The cells were stimulated for approximately 6 days with a medium change every 2 days until the mature osteoclasts were formed. Then, the osteoclast differentiation was evaluated by a TRAP staining assay, and the expression of osteoclast-related genes was examined with qRT-PCR. The cells were washed twice with PBS, fixed with 4% paraformaldehyde for 20 minutes, and stained for TRAP (SigmaAldrich, St Louis, MO, USA). TRAP-positive osteoclasts with more than three nuclei were counted under an inverted microscope.

### RNA isolation and qRT-PCR analysis

The BMSCs were seeded at 2 × 10^4^ cells/cm^2^ in 6-well plates and cultured in OBM with different concentrations of PSP. After being cultured at these intervals, the total RNA was extracted using RNAiso plus reagent (TakaraBio, Dalian, China) according to the manufacturer’s instructions. The RNA concentration and purity were evaluated using an ultraviolet spectrophotometer. The cDNA (20 μL) was synthesised from 2.5 μg RNA using a RevertAid First Strand cDNA Synthesis Kit (Thermo Fisher Scientific, USA). Expression of ALP, COL I, Runx2, and OCN was quantified with a quantitative real-time PCR, carried out in a 20 μL SYBR Green PCR master mix (Roche, Mannheim, Germany) by an Applied Biosystems (ABI) 7500 Real-Time PCR System. The relative expression of gene-specific products was analysed using the 2^−ΔΔCt^ method and normalised to the corresponding β-actin values. All results were confirmed by repeating the experiment 3 times. The expression of osteoclast-related genes (TRAP, MMP-9, CtsK, and NFATc1) was determined in the same manner. The primers, all designed and synthesised by Sangon (Shanghai, China) Biological Engineering Technology and Service Co, LTD, were as follows:

β-actin (forward: 5′-ATGGCTGGGGTGTTGAAGGT-3′ and reverse: 5′-ATCTGGCACCACACCTTCTACAA-3′), ALP (forward: 5′-CCAACTCTTTTGTGCCAGAGA-3′ and reverse: 5′-GGCTACATTGGTGTTGAGCTTTT-3′), COL I (forward: 5′-CCCAGAGTGGAACAGCGATT -3′ and reverse: 5′-ATGAGTTCTTCGCTGGGGTG-3), Runx2 (forward: 5′-TTCTCCAACCCACGAATGCAC-3′ and reverse: 5′-CAGGTACGTGTGGTAGTGAGT-3′), OCN (forward: 5′-GAGGGCAATAAGGTAGTGAACAGA-3′ and reverse: 5′-AAGCCATACTGGTTTGATAGCTCG-3′), TRAP (forward: 5′-GGTATGTGCTGGCTGGAAAC-3′ and reverse: 5′-GGTAGTAAGGGCTGGGGAAG-3′), MMP-9 (forward: 5′-CCCCAAAGACCTGAAAACCT-3′ and reverse: 5′-GCTTCTCTCCCATCATCTGG-3′), CtsK (forward: 5′-GTGTTGGTGGTGGGCTATG-3′ and reverse: 5′-GCAGGCGTTGTTCTTATTCC-3′), NFATc1 (forward: 5′-TGTCCAACACCAAAGTCCTG-3′ and reverse: 5′-TCTTCCTCCCGATGTCTGTC-3′), β-catenin (forward: 5′-GCGTGGACAATGGCTACTCAAG-3′and reverse: 5′-GTCATTGCA TACTGCCCGTCAA-3′).

### Protein extracts and WB analysis

As previously described, the BMSCs were cultured in OBM with different concentrations of PSP. After treatment, the total protein extracts were removed using a Lysis Buffer (Beyotime Biotechnology, Shanghai, China) containing 1 mM PMSF. Cytoplasmic and nuclear protein extracts were separated using a Nuclear and Cytoplasmic Protein Extraction Kit (KeyGEN BioTECH, Nanjing, China) according to the manufacturer’s instructions. The protein content was quantitated using the BCA method. After degeneration, equal amounts (40 μg) of protein samples were separated by 10% SDS-PAGE and then electro-transferred onto nitrocellulose membranes. After blocking the membranes with 5% non-fat milk for 1 hour, the membranes were incubated with the following primary antibodies: anti-β-catenin (1:1000), anti-β-actin (1:1000), anti-GAPDH (1:5000), anti-Histone H3 (1:500), anti-Smad1/5/8 (1:500) and anti-p-Smad1/5/8 (1:1000) at 4 °C overnight, and then the secondary antibody (1:500) for 1 hour at room temperature away from any light. The membranes were swept using an Odyssey Infrared Imaging Scanner System (LI-COR Bioscience, USA), and the greyscales of the images were analysed with Image J software.

### Immunohistochemistry staining

Immunohistochemistry staining for COL I and OCN of the BMSCs treated with PSP was performed using a standard, indirect three-step immunoperoxidase technique. Cold methanol was added onto the cells for fixation; the cells were then incubated with 3% hydrogen peroxide for 10 minutes to block the endogenous peroxidase. The primary antibody (rabbit anti-COL I 1:500, rabbit anti-OCN 1:600) was applied overnight. Next, DAB served as the chromogen, and hematoxylin served as the counterstain. Samples treated with a phosphate buffer served as the negative control. The positivity rate was calculated under 200 × magnifications by dividing the number of positive cells by the total number of cells counted in five random visual fields and expressed as a percentage. Photographs were taken with a digital image-capture system.

### Immunofluorescence assay for β-catenin nuclear translocation

The BMSCs were plated at a density of 2 × 10^4^ cells/well on sterile glass coverslips pre-coated with 100 mg/L poly-D-lysine in 24-well plates; the cells were treated in triplicate with PSP at a concentration of 25 mg/L for 72 hours once the monolayer had reached 50% confluence. For immunofluorescence analysis, the cells were firstly fixed and then permeabilised with 0.1% Triton X-100/PBS. Next, the endogenous peroxidase were effectively eliminated with 3% H_2_O_2_, and the non-specific staining was blocked with normal goat serum. Glass coverslips were gently placed on the top of a 1.5 ml EP tube box. The bottom of this box contained distilled water to keep it moist. The cell side was facing up in the box, and the cells were incubated overnight at 4 °C with 50 μl/slip anti-β-catenin (1:200). After washes in PBS, the slips were incubated with biotin-conjugated goat anti-rabbit IgG (1:100) for 1 hour. This was followed by SABC-Cy3 (1:100) in the dark for 15 minutes and subsequently stained with 10 μg/ml Hoechst 33258 for 20 minutes. After the cell sides facing down were sealed with an anti-fade-mounting medium, the images were obtained using a laser scanning confocal microscope (Nikon A1) at a magnification of 40 × (oil lens).

### Transient transfection and dual-luciferase reporter assays

The BMSCs were plated at a density of 1 × 10^4^ cells/well into a 96-well plate for 24 hours prior to plasmid infection. At 60–70% confluence, the cells were co-transfected with 100 ng luciferase reporter plasmid (TOPFlash or FOPFlash) (Millipore Corporation, Temecula, CA, USA) and 20 ng pRL-TK vector (Promega Corporation, Madison, USA) according to the Lipofectamine 3000 Reagent protocol (Invitrogen, USA). Scale volumes were proportional for additional wells. The infected cells were placed in an incubator at 37 °C with 5% CO_2_. After being transfected for 6–8 hours, the plasmid–lipid mixtures were removed from the plate wells and replaced with various concentrations of PSP as described previously. After 48 hours, the cells were harvested and lysed, and the firefly luciferase and Renilla luciferase activities were measured using a single-tube chemiluminescence detector (Berthold Lumat LB9508, Niedersachsen, Germany) according to the Dual-Luciferase Reporter Assay System protocol (Promega Corporation, Madison, USA). The relative luciferase activity, as the ratio between firefly luciferase and Renilla luciferase, reflected the activation of transcription factors of a Wnt/β-catenin signalling pathway in the BMSCs. Renilla-luciferase was used to normalise the transfection efficiency. The experiments were performed independently in triplicate.

### *In vivo* murine calvarial model of LPS-induced osteolysis

A total of 24 8-week old C57BL/J6 mice divided into 3 groups: phosphate-buffered saline (PBS; control), LPS (5mg/kg body weight; Sigma-Aldrich) and LPS + PSP (15 g/kg body weight). Injections of LPS, LPS and PSP or PBS were made into the subcutaneous tissue overlying the middle suture of calvaria. Seven days after the first LPS injections mice were sacrificed and calvarias dissected and fixed in 4% paraformaldehyde for 1 day at 4 °C. No adverse affects or fatalities were noted.

Three-dimensional reconstructions of whole calvaria were obtained from images using a high-resolution micro-CT (μCT) scanner (Skyscan 1176; Skyscan; Aartselaar, Belgium). Image acquisition was conducted at a voltage of 50 kV, an isotropic pixel size of 14.4 μm (1024 × 1024 pixel image matrix), current of 800 μA, and with a 0.75-mm-thick aluminum filter for beam hardening reduction. A square region of interest (ROI) around the midline suture was chosen for further qualitative and quantitative analysis. The BV/TV (bone volume/tissue volume), Tb.N (trabecular number), Tb.Sp (trabecular separation) and Tb.Th (trabecular thickness) were measured. For histological and histomorphometric analysis, the calvarial samples were decalcified in 10% EDTA (pH 7.4) at 4 °C for 2 weeks, followed by paraffin embedding. Histological sections were prepared for TRAP and H&E staining and examined at 40X and 200X magnification (Nikon Eclipse E200 microscope). The number of TRAP-positive multinucleated osteoclasts per field and Erosion Area(%) were examined in each sample using Image-Pro Plus software (Media Cybernetics, Bethesda, MD, USA).

### Statistical analysis

All quantitative data were expressed as a mean ± SD and were analysed by a one-way analysis of variance (ANOVA) using SPSS16.0 statistical software. Comparisons among groups were assessed using an LSD test and Dunnett (double-sided) test. Significant differences were classified as *p < 0.05 and **p < 0.01. Bar graphs were drawn by using GraphPad Prism v5.0 software.

## Additional Information

**How to cite this article**: Du, L. *et al*. Polygonatum sibiricum polysaccharide inhibits osteoporosis by promoting osteoblast formation and blocking osteoclastogenesis through Wnt/β-catenin signalling pathway. *Sci. Rep.* **6**, 32261; doi: 10.1038/srep32261 (2016).
